# Perception of pathogenic or beneficial bacteria and their evasion of host immunity: pattern recognition receptors in the frontline

**DOI:** 10.3389/fpls.2015.00219

**Published:** 2015-04-08

**Authors:** Lucie Trdá, Freddy Boutrot, Justine Claverie, Daphnée Brulé, Stephan Dorey, Benoit Poinssot

**Affiliations:** ^1^Université de Bourgogne, UMR 1347 Agroécologie, Pôle Interactions Plantes Micro-organismes - ERL CNRS 6300Dijon, France; ^2^Laboratory of Pathological Plant Physiology, Institute of Experimental Botany, Academy of Sciences of Czech RepublicPrague, Czech Republic; ^3^The Sainsbury Laboratory, Norwich Research ParkNorwich, UK; ^4^Laboratoire Stress, Défenses et Reproduction des Plantes, URVVC EA 4707, Université de Reims Champagne-ArdenneReims, France

**Keywords:** plant–microbe interactions, innate immunity, evasion, MAMP, PRR, flg22, FLS2, LysM

## Abstract

Plants are continuously monitoring the presence of microorganisms to establish an adapted response. Plants commonly use pattern recognition receptors (PRRs) to perceive microbe- or pathogen-associated molecular patterns (MAMPs/PAMPs) which are microorganism molecular signatures. Located at the plant plasma membrane, the PRRs are generally receptor-like kinases (RLKs) or receptor-like proteins (RLPs). MAMP detection will lead to the establishment of a plant defense program called MAMP-triggered immunity (MTI). In this review, we overview the RLKs and RLPs that assure early recognition and control of pathogenic or beneficial bacteria. We also highlight the crucial function of PRRs during plant-microbe interactions, with a special emphasis on the receptors of the bacterial flagellin and peptidoglycan. In addition, we discuss the multiple strategies used by bacteria to evade PRR-mediated recognition.

## Introduction

Plants are an attractive source of nutrients and life environment for many bacteria. They are colonized by pathogenic bacteria resulting in various diseases, but also by non-pathogenic soil and epiphyte bacteria providing beneficial effects on plant growth or stress resistance. Plants form symbiosis with strictly biotrophic nitrogen-fixing *Rhizobium* or mutualistic interaction with plant growth-promoting rhizobacteria (PGPR) (Lugtenberg and Kamilova, 2009; Oldroyd et al., 2011). Both *Rhizobium* and PGPR were described to improve plant growth and enhance broad-spectrum resistance to biotic and abiotic stresses (Lugtenberg and Kamilova, 2009; Beardon et al., 2014; Pieterse et al., 2014). Pathogenic as well as beneficial bacteria are initially recognized as harmful invaders in order to limit the bacterial spread (Pel and Pieterse, 2013). This recognition is assured by an efficient plant immune system, highly similar to animal innate immunity.

At the frontline, plants possess plasma-membrane localized pattern recognition receptors (PRRs) that recognize microbe/pathogen-associated molecular patterns (MAMPs/PAMPs). These conserved signatures are part of crucial microbial structures, such as cell walls or motility organs (Boller and Felix, 2009; Newman et al., 2013). Specific PRRs also detect host-derived damage-associated molecular patterns (DAMPs), which are plant cell wall fragments or peptides produced as a consequence of mechanical injuries or enzymatic microbial activities (Boller and Felix, 2009; Monaghan and Zipfel, 2012; Newman et al., 2013; Savatin et al., 2014). PRR-mediated microbe sensing induces a broad variety of defense responses commonly referred to as MAMP- or PAMP-triggered immunity (MTI/PTI) (Zipfel, 2014). MTI is a defense program with complex early signaling events leading to the massive transcriptional reprogramming (Boller and Felix, 2009; Liu et al., 2014; Tsuda and Somssich, 2015) that initiates defense responses such as stomatal closure, cell wall strengthening, and production of antimicrobial compounds (Supplementary Figure S1). However, successful pathogens evolved to suppress or interfere with the MTI responses by secreting different compounds such as effectors, proteases or toxins, resulting in facilitated host colonization (Jones and Dangl, 2006). In an ongoing arms-race between the host and attacking microorganism, plants evolved host-specific intracellular receptor (R) proteins to detect the presence or activities of effectors and to initiate a defense program in the so-called effector-triggered immunity (ETI) (Jones and Dangl, 2006; Cui et al., 2014; Wu et al., 2014).

In this review, we summarize our current knowledge on PRRs-mediated recognition of bacteria, the importance of this crucial monitoring step in the context of plant disease and establishment of beneficial interaction. We also address the question of PRR evolution and the species-specific recognitions. The main focus is on the perception of the extensively-studied MAMPs flagellin and peptidoglycan.

## PRRs: a highly diverse family of receptors

Plant PRRs are plasma membrane-localized receptor-like kinases (RLKs) or receptor-like proteins (RLPs) with an extracellular domain for MAMP recognition. The transmembrane RLKs contain a cytosolic serine/threonine kinase domain, while RLPs can be either glycosylphosphatidylinositol (GPI)-anchored or transmembrane proteins lacking a kinase domain. The extracellular domains of RLKs and RLPs, which confer ligand specificities, are organized into subfamilies according to domain composition (Shiu and Bleecker, 2001). While lysine motifs (LysM) or lectin motifs are common ectodomains in RLKs and RLPs, the leucine-rich repeat (LRR) extracellular motif are the most represented in plants like Arabidopsis and rice (Shiu et al., 2004), tomato (Sakamoto et al., 2012), or soybean (Liu et al., 2015). LRR domains are widespread among living organisms where they provide a structural framework for protein–protein interactions (Ng et al., 2011). In plants, LRR containing proteins are most often associated to signal transduction and immunity, and several studies have revealed their specific binding to proteinaceous microbial ligands (Boller and Felix, 2009; Monaghan and Zipfel, 2012).

The LRR-RLK FLAGELLIN-SENSITIVE 2 (FLS2) is among the best characterized plant PRRs. FLS2 detects bacterial invasion by recognition and direct binding of flagellin *via* its flg22 epitope (Gomez-Gomez and Boller, 2000; Chinchilla et al., 2006; Sun et al., 2013). FLS2 orthologs are found in other plant species including tomato (Robatzek et al., 2007), rice (Takai et al., 2008), or grapevine (Trdá et al., 2014). Other PRRs involved in the monitoring of bacteria include the LRR-RLK ELONGATION FACTOR-TU (EF-Tu) RECEPTOR (EFR), which perceives bacterial EF-Tu and its peptide epitope elf18 (Zipfel et al., 2006), and LysM-containing RLKs and RLPs, which mediate the recognition of *N*-acetylglucosamine (GlcNAc)-containing ligands present on microbial surface, such as bacterial peptidoglycans (PGNs) but also fungal chitin (Gust et al., 2012). Chitin-related PRR system is well-studied in both dicots and monocots. Chitin hepta- or octamers are recognized by the LysM-RLK CHITIN ELICITOR RECEPTOR KINASE 1 (CERK1) and LYSIN MOTIF-CONTAINING RECEPTOR-LIKE KINASE5 (LYK5) in *Arabidopsis thaliana* (Miya et al., 2007; Wan et al., 2008; Petutschnig et al., 2010; Liu et al., 2012b; Cao et al., 2014) and by the complex OsCERK1/CHITIN ELICITOR-BINDING PROTEIN (CEBiP) in rice (Kaku et al., 2006; Miya et al., 2007; Shimizu et al., 2010). PGN perception involves CERK1 and LysM-RLPs, in Arabidopsis and rice (Willmann et al., 2011; Ao et al., 2014).

Among other identified PRRs are several LRR-RLPs, like the tomato LeEix1 and LeEix2, which bind fungal-derived ethylene-inducing xylanases (Ron and Avni, 2004), the tomato receptor Ve1, which recognizes the protein Ave1 from *Verticillium* fungi (de Jonge et al., 2012), the Arabidopsis AtRLP1/ReMAX, which detects a proteinaceous MAMP from *Xanthomonas* (Jehle et al., 2013), the Arabidopsis AtRLP30 detecting the proteinaceous elicitor SCLEROTINIA CULTURE FILTRATE ELICITOR1 (SCFE1) purified from the axenic culture filtrate of *Sclerotinia sclerotiorum* (Zhang et al., 2013), and RESPONSIVENESS TO BOTRYTIS POLYGALACTURONASE 1 (RBPG1/AtRLP42), which recognizes fungal endopolygalacturonases from *Botrytis cinerea* or *Aspergillus niger* (Zhang et al., 2014).

Following activation, PRRs are recruited to molecular complexes where they initiate downstream signaling. Functionality of many RLK- or RLP-PRRs then depends on heterodimerization with regulatory RLKs which improve ligand recognition and control intracellular signaling through autophosphorylation or transphosphorylation events (Monaghan and Zipfel, 2012; Böhm et al., 2014; Han et al., 2014). BRASSINOSTEROID INSENSITIVE 1 (BRI1)-ASSOCIATED KINASE 1 (BAK1)/SOMATIC EMBRYOGENESIS RECEPTOR-LIKE KINASE 3 (SERK3) is one of the key regulatory RLK assuring signaling for several RLK-PRRs including FLS2 (Chinchilla et al., 2007; Heese et al., 2007; Sun et al., 2013), and EFR (Roux et al., 2011). BAK1 and its homologs are also required for the functionality of RLP-PRRs such as Ve1 (Fradin et al., 2009), LeEix1 (Bar et al., 2010), and RLP30 (Zhang et al., 2013). Many LRR-RLPs also interact with SUPPRESSOR OF BIR1-1 (SOBIR1) which seems to function as an universal adaptor for different receptors (Liebrand et al., 2013; Zhang et al., 2013; Gust and Felix, 2014). Similarly, the LysM-RLK CERK1 appears to have regulatory functions for others RLKs such as LYK5 to control chitin perception in Arabidopsis (Cao et al., 2014), or for RLPs such as CEBiP to sense chitin in rice (Shimizu et al., 2010) or LYSM DOMAIN GPI-ANCHORED PROTEIN1 and 3 (LYM1 and LYM3) to control PGN recognition in Arabidopsis (Willmann et al., 2011).

## PRRs at a frontline during the interactions with bacteria

Upon interaction with bacteria, plants activate immune system following the detection of a variety of MAMPs like flagellin, EF-Tu, PGN, or lipopolysaccharides (LPS) (Boller and Felix, 2009) (Supplementary Figure S1). Different works highlight the relevance of the PRR-mediated MTI in plant disease resistance against bacteria. Firstly, the exogenous applications of bacteria-derived MAMPs (Wiesel et al., 2014; Burketová et al., 2015) or living bacteria (Manikandan and Raguchander, 2014) can enhance plant resistance against bacterial diseases. Secondly, studies using knock-out or silenced mutant plants for given PRRs reveal their contribution in the context of plant bacterial disease (Table 1). Loss-of-function approaches are mainly studied in Arabidopsis upon infection with *Pseudomonas syringae* (notably the pathovar *tomato* (*Pto*) DC3000), a foliar pathogen of tomato that also infects Arabidopsis. Gain-of-function analyses also reveal that PRR transfer is able to confer resistance. The expression of *AtEFR* in *Nicotiana benthamiana*, tomato or rice plants results in increased resistance to *P. syringae* pv. *tabaci* (*Pta*), tumorigenic *Agrobacterium tumefaciens*, *Ralstonia solanacearum* and *Xanthomonas oryzae* pv. *oryzae (Xoo)* after binding with elf18 epitope (Zipfel et al., 2006; Lacombe et al., 2010; Lu et al., 2015). Similarly the *AtEFR* transfer in wheat results in enhanced resistance against the cereal bacterial pathogen *Pseudomonas syringae* pv. *oryzae* demonstrating that PRRs can also be successfully transferred from dicot to monocot species (Schoonbeek et al., 2015). While every microbe will expose several MAMPs to host, many of these MAMPs induce comparable signaling events that converge to a common defense response (Wan et al., 2008; Boller and Felix, 2009). Nevertheless, several MAMP/PRR pairs are individually contributing to bacterial resistance (Table 1), indicating a potential quantitative contributions of certain MAMPs or an absence of redundancy in eliciting activities.

**Table 1:** **RLKs and RLPs involved in basal resistance against bacteria**.

**RLK/RLP**	**Family**	**Plant**	**MAMP**	**Bacteria**	**References**
**PATHOGENIC BACTERIA**					
FLS2	LRR-RLK	*A. thaliana*	Flagellin	*Pto* DC3000	Zipfel et al., 2004; Xiang et al., 2008
				*Psp* RW60	de Torres et al., 2006
				*Pto* DC3000, *Pto* DC3000 *COR*-, *Pto* DC3000 *Δ AvrPto/Δ AvrPtoB*, *Pta* 6605	Nekrasov et al., 2009
				*Pta* 6605, *Psg* race4, *Pto* T1	Ishiga et al., 2011
				*Pto* DC3000, *Pto* DC3000 *hrcC*-, Pto DC3000 *Δ HopU1*	Nicaise et al., 2013
		*N. benthamiana*		*Pta*, *Pto* T1, *Pto* DC3000, *Pto* DC3000 *hrcC*-	Hann and Rathjen, 2007
EFR	LRR-RLK	*A. thaliana*	EF-Tu	*Agrobacterium tumefaciens*	Zipfel et al., 2006
				*Pto* DC3000 *Δ AvrPto/Δ AvrPtoB*	Nekrasov et al., 2009
XA21^a^	LRR-RLK	*O. sativa*		*Xoo* PX061	Zhao et al., 2009
BAK1/SERK3	LRR-RLK	*A. thaliana*		*Pto* DC3000, *Pto* DC3000 *hrcC*-, *Pta* 6605	Roux et al., 2011
NbSERK3	LRR-RLK	*N. benthamiana*		*Pto* DC3000, *Pto* DC3000 *hrcC*-, *Pta* 6605	Heese et al., 2007
OsSERK2	LRR-RLK	*O. sativa*		*Xoo* PXO99AZ	Chen et al., 2014b
IOS1	LRR-RLK	*A. thaliana*		*Pto* DC3000, *Psm* ES4326	Chen et al., 2014a
BIR2^b^	LRR-RLK	*A. thaliana*		*Pto* DC3000	Halter et al., 2014
LIK1^b^	LRR-RLK	*A. thaliana*		*Pto* DC3000	Le et al., 2014
PSKR1^b^	LRR-RLK	*A. thaliana*	PSK	*A. tumefaciens* C58 nocc	Loivamäki et al., 2010
				*Pto* DC3000	Mosher et al., 2013
RLP30	LRR-RLP	*A. thaliana*	SCFE1	*Psp* 1448A	Wang et al., 2008; Zhang et al., 2013
CERK1	LysM-RLK	*A. thaliana*	GlcNAc	*Pto* DC3000, *Pto* DC3000 *hrcC*-, *Pto* DC3000 *Δ AvrPtoB*	Gimenez-Ibanez et al., 2009; Willmann et al., 2011; Wan et al., 2012
Bti9, SILyk13	LysM-RLK	*S. lycopersicum*		*Pto* DC3000 *Δ AvrPto/Δ AvrPtoB/Δ HopQ1- 1/Δ FliC*	Zeng et al., 2012
LYK1	LysM-RLP	*A. thaliana*		*Pto* DC3000, *Pto* DC3000 *hrcC*-	Wan et al., 2012
LYK3^b^	LysM-RLP	*A. thaliana*		*Pcc* DSMZ 30169	Paparella et al., 2014
LYK4	LysM-RLP	*A. thaliana*		*Pto* DC3000	Wan et al., 2012
LYM1	LysM-RLP	*A. thaliana*	PGN	*Pto* DC3000	Willmann et al., 2011
LYM3	LysM-RLP	*A. thaliana*	PGN	*Pto* DC3000, *Pto* DC3000 *hrcC*-, *Pto* DC3000 *Δ AvrPto/Δ AvrPtoB*	Willmann et al., 2011
LYP4, LYP6	LysM-RLP	*O. sativa*		*Xoc* GDx, *Xoo* GD4	Liu et al., 2012a
LORE	G-Lec-RLK	*A. thaliana*	LPS	*Pto* DC3000	Ranf et al., 2015
CaMBL1	G-Lec-RLP	*C. annuum*		*Xcv* Ds1*, Xcv* Bv5-4a	Hwang and Hwang, 2011
LecRK-IV.4, LecRK-S.1, LecRK-S.4	L-Lec-RLK	*A. thaliana*		*Pto* DC3000	Wang et al., 2014
LecRK-V.5^b^	L-Lec-RLK	*A. thaliana*		*Pto* DC3000, *Pcc* WPP14	Arnaud et al., 2012; Desclos-Theveniau et al., 2012
LecRK-VI.2	L-Lec-RLK	*A. thaliana*		*Pto* DC3000, *Pto* DC3000 COR-, *Pcc* SCC1	Singh et al., 2012
CRK13^a^	DUF26-RLK	*A. thaliana*		*Pto* DC3000	Acharya et al., 2007
CRK20^b^	DUF26-RLK	*A. thaliana*		*Pto* DC3000	Ederli et al., 2011
**PLANT GROWTH-PROMOTING RHIZOBACTERIA (PGPR)**					
PRK4^b^	LRR-RLK	*A. thaliana*		*Bacillus subtilis* FB17	Lakshmanan et al., 2013
WAK3^b^	WAK-RLK	*A. thaliana*		*B. subtilis* FB17	Lakshmanan et al., 2013
**SYMBIOTIC BACTERIA**					
NFR1	LysM-RLK	*L. japonicus*	Nod factor	*Mesorhizobium loti*	Radutoiu et al., 2003
NFR5	LysM-RLK	*L. japonicus*	Nod factor	*M. loti*	Madsen et al., 2003
SYMRK	LysM-RLK	*L. japonicus*		*M. loti*	Stracke et al., 2002
LYK3	LysM-RLK	*M. truncatula*		*Sinorhizobium meliloti* GMI5622	Limpens et al., 2003
NFP	LysM-RLK	*M. truncatula*	Nod factor	*S. meliloti* GMI5622, *S. meliloti* SM2011	Arrighi et al., 2006
DMI2/NORK	LysM-RLK	*M. truncatula*		*S. meliloti*, *S. meliloti* GMI5622	Endre et al., 2002; Limpens et al., 2005
PaNFP	LysM-RLK	*P. andersonii*	Nod factor	*Sinorhizobium sp*. NGR234	Op den Camp et al., 2011

a *Determined only by gain-of-function analysis*.

b *Bacterial growth enhanced in the loss-of-function mutant. LRR, Leucine-rich repeat; LysM, lysine motif; G-Lec, G-type lectin; L-Lec, L-type lectin; DUF26, domain of unknown function 26; WAK, wall-associated kinase*.

*Pcc, Pectobacterium carotovorum subsp. carotovorum; Psg, Pseudomonas syringae pv. glycinea; Psm, Pseudomonas syringae pv. maculicola; Psp, Pseudomonas syringae pv. phaseolicola; Pta, Pseudomonas syringae pv. tabaci; Pto, Pseudomonas syringae pv. tomato; Xcv, Xanthomonas campestris pv. vesicatoria; Xoc, X. oryzae pv. oryzicola; Xoo, X. oryzae pv. oryzae*.

### Involvement of the flagellin/FLS2 perception system in plant-bacteria interaction

**Figure 1:**
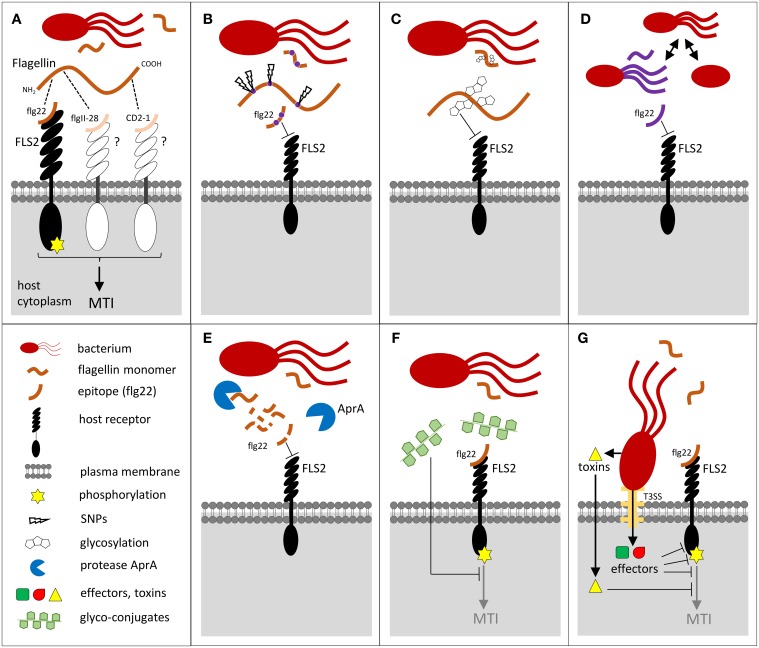
**Potential bacterial strategies employed to evade flagellin recognition *via* FLS2 upon bacterial invasion in plant tissues. (A)** Flagellin monomers are recognized by FLS2 *via* the flg22 epitope, or possibly by other putative receptors detecting the epitopes flgII-28 in Solanaceae and CD2-1 in rice. The ligand binding triggers receptor kinase phosphorylation and activates defense responses leading to MAMP-triggered immunity (MTI). **(B–G)** Evasion strategies that hamper FLS2 recognition: **(B)** SNPs within the gene encoding the flagellin epitopes, **(C)** flagellin post-translational modifications such as glycosylation, **(D)** several bacterial pathogens are aflagellated, loose flagellin upon colonization, or express alternative flagellins, **(E)** alkaline protease AprA degrades flagellin. **(F)** Flagellin-mediated MTI is also inhibited by glyco-conjugates such as extracellular polysaccharides or cyclic glucan *via* yet poorly understood mechanisms, or **(G)** by bacterial effectors injected to plant cell by Type-III secretion system (T3SS), or by toxins.

Upon *P. syringae* infection, the FLS2-mediated sensing of flagellin is important to restrict the bacterial invasion both in Arabidopsis (Zipfel et al., 2004; Zhang et al., 2007; Xiang et al., 2008; Zeng and He, 2010) and in *N. benthamiana* (Hann and Rathjen, 2007). Pretreatment with the immunogenic flagellin-derived flg22 epitope induce MTI (Figure 1) and trigger protection against virulent pathogens such as *Pto* in Arabidopsis (Zipfel et al., 2004). Flagellin perception results in stomatal closure during the initial stage of invasion through stomata (Zeng and He, 2010). The highest expression of *FLS2* maps to tissues vulnerable for bacterial entry, such as stomata, hydathodes, and lateral roots (Beck et al., 2014) and is also correlated with the limitation of *Pto* colonization (Vetter et al., 2012). Accordingly, the enhanced susceptibility of the *fls2* mutant toward *Pto* is observed when plants are infected by inoculum spray or dipping, but not with apoplast-infiltrated inoculum, suggesting that flagellin perception restricts bacterial invasion at an early step but does not play a major role in post-entry defenses (Zipfel et al., 2004; Zeng and He, 2010). However, in the root system, the intensity of the immune responses does not always correlate with the expression level of the FLS2 receptor, but rather depends on the expressing tissue (Wyrsch et al., 2015).

Flagellin from *Xanthomonas campestris* pv. *campestris* (*Xcc*), an important vascular pathogen of Brassicaceae, elicits MTI in Arabidopsis in a strain-specific way. Pretreatment with *Xcc*-derived flagellin also restricts *Xcc* infection of Arabidopsis plants (Sun et al., 2006). However, the flagellin immunogenicity did not limit the growth of virulent isogenic *Xcc* strain in Arabidopsis leaves, suggesting that *Xcc* evades or interferes with PRR-mediated immunity (Sun et al., 2006).

FLS2 can also detect flagellins of beneficial microbes to initiate plant defense responses, though to our knowledge the mechanisms, which allow these bacteria to colonize plants, are still limited. For instance, the flagellin from the PGPR *Pseudomonas fluorescens* (WCS374 and WCS417), and *Pseudomonas putida* (WCS358 and KT2440) induces some innate immune responses in tobacco cells or maize plants (van Loon et al., 2008; Planchamp et al., 2014). Flagellin and flg22 from the endophytic PGPR *Burkholderia phytofirmans* PsJN trigger MTI in an FLS2-dependent manner in Arabidopsis and to a lesser extent in grapevine (Trdá et al., 2014). *Bacillus*-induced stomatal closure is abolished in the Arabidopsis *fls2* mutant plant, supporting that flagellin perception also contributes to this rhizobacteria-mediated defense response (Kumar et al., 2012), and further supporting the biological function of flagellin perception in roots (Millet et al., 2010; Beck et al., 2014; Wyrsch et al., 2015). Flg22-triggered defense delays nodule organogenesis in the early symbiotic establishment between *Lotus japonicus* and *Sinorhizobium meliloti* (Lopez-Gomez et al., 2012). However, no effect of flg22 is observed once the symbiosis is established, probably because of secreted MTI-suppressing factors (Lopez-Gomez et al., 2012; Zamioudis and Pieterse, 2012). Interestingly, the *LjFLS2* expression is down-regulated in nodules (Lopez-Gomez et al., 2012), even though the flagellin of *S. meliloti* is not immunogenic.

### Involvement of the LysM perception systems in plant–bacteria interaction

PGN is another MAMP present in bacterial cell walls. PGN consists of heteropolymeric chains of *N*-acetylglucosamine (GlcNAc) and *N*-acetylmuramic acid (MurNAc) crosslinked with a short peptide. PGN is structurally related to chitin and plant symbiont-secreted lipochitooligosaccharides. The perception of PGN from both Gram-positive and Gram-negative bacteria in Arabidopsis requires two LysM-RLPs, AtLYM1 and AtLYM3, which specifically bind PGN, and the LysM-RLK CERK1 (Gimenez-Ibanez et al., 2009; Willmann et al., 2011). The PGN sensing system is similar in rice, involving the LysM-RLK, OsCERK1, and OsLYP4 and OsLYP6, the LYM1 and LYM3 homologs (Liu et al., 2012a; Ao et al., 2014; Kouzai et al., 2014). The PGN-sensing PRRs are involved in the bacterial resistance, as the *lym1* and *lym3* mutants, insensitive to PGN, exhibit hypersusceptibility to infection with virulent *Pto* (Willmann et al., 2011). The silencing of *OsLYP4* or *OsLYP6* also leads to compromised resistance to the bacterial blight of rice caused by *X. oryzae* (Liu et al., 2012a). Loss of AtCERK1 results in increased susceptibility to bacterial infection caused by *Pto* DC3000 in Arabidopsis (Gimenez-Ibanez et al., 2009). Currently, the role of OsCERK1 in disease resistance is not clear. Remarkably, the components of GlcNAc sensing are independent from the flagellin sensing system requiring LRR-RLK complexes (Böhm et al., 2014).

LysM-containing PRR-like proteins are also implicated in the detection of rhizobia. Upon interaction, rhizobia secrete lipochitooligosaccharidic nodulation (Nod) factors (NFs). The structure of rhizobial Nod factors varies according to the strain and determines the host specificity (Radutoiu et al., 2007). In *L. japonicus*, the LysM-RLKs NF RECEPTOR 1 (LjNFR1) and LjNFR5 (Broghammer et al., 2012) recognize and directly bind NFs. Their homologs mediate NF sensing in other legumes such as MtNFP (Arrighi et al., 2006) and MtLYK3 (Limpens et al., 2003; Smit et al., 2007) in *Medicago truncatula*, or PsSym37 and PsSym10 in *Pisum sativum* (Gust et al., 2012). NF recognition is crucial for the establishment of symbiosis between a host plant and rhizobia (Geurts et al., 2005; Liu et al., 2007; Van Wees et al., 2008; Pieterse et al., 2014). Even though Arabidopsis does not form symbioses, it recognizes Nod factors *via* the LysM-RLK AtLYK3 (Liang et al., 2013). This recognition results in a strong suppression of flg22-induced immune responses and resistance to *Pto* (Liang et al., 2013). Interestingly, MtNFP, together with another LysM-RLK, MtLYR3, also seems to be involved in the perception of Myc factors, which are symbiosis-mediating signals in the arbuscular mycorrhiza (Maillet et al., 2011; Czaja et al., 2012; Fliegmann et al., 2013). Therefore, it seems that plants use overlapping systems to detect fungal and bacterial stimuli in both pathogenic and beneficial interactions.

## The evasion of PRR-mediated immunity by bacteria: evolution of MAMPs as a crucial process

In animals, ε-proteobacteria, including the important pathogens *Helicobacter pylori* or *Campylobacter jejuni*, evades the TOLL-LIKE RECEPTOR 5 (TLR5) flagellin recognition system by mutating their entire flagellin recognition site (Andersen-Nissen et al., 2005; Broz and Monack, 2013). In plants, the immunogenic epitopes elf18 and flg22 also diversified among different bacteria species and strains (Sun et al., 2006; Cai et al., 2011), and with a higher rate than the non-immunogenic protein parts (McCann et al., 2012). For pathogens such as *Xcc*, which are co-evolving with Brassicaceae, strains presenting a single amino acid polymorphism in flg22 can completely abolish the *Xcc* flagellin eliciting activity in Arabidopsis (Sun et al., 2006) (Figure 1B). Similar results were recently observed in rice, where *X. oryzae* pv. *oryzae (Xoo)* and pv. *oryzicola* (*Xoc*) evade rice FLS2 recognition with flg22 site mutations (Wang et al., 2015). The flg22 epitopes derived from *A. tumefaciens*, *S. meliloti*, and *R. solanacearum* are highly divergent and also escape recognition by Arabidopsis or tomato (Felix et al., 1999; Bauer et al., 2001; Pfund et al., 2004) and the flagellin of *S. meliloti* is not recognized in the host legume *L. japonicus* (Lopez-Gomez et al., 2012). It was suggested that alteration in the flg22 sequence of the PGPR *B. phytofirmans* might be a successful adaptation of this bacteria to avoid recognition by VvFLS2 in the grapevine host (Trdá et al., 2014). Several studies have also demonstrated that perception of a same flg22 peptide varies quantitatively in different plant species and ecotypes (Albert et al., 2010; Vetter et al., 2012; Veluchamy et al., 2014). Overall, these differences in responsiveness could reflect FLS2 co-evolution driving the detection of flagellin alleles of ecologically relevant microbial strains.

While mutations within the flg22 epitope can lead to complete MTI evasion, an additional flgII-28 epitope has recently been identified within the flagellin protein of *Pto* isolates. This epitope is active in Solanaceae but not in Arabidopsis, and is also under a strong selective pressure (Cai et al., 2011; Clarke et al., 2013) (Figure 1A). Over the last 30 years, the ancestral *flgII-28* allele almost completely disappeared from the worldwide population of *Pto* and was replaced by a novel variant with reduced capacity to elicit a plant defense response (Cai et al., 2011). Surprisingly, an eliciting activity has also recently been discovered in CD2-1, a third region in the flagellin protein from the rice avirulent *Acidovorax avenae* (Katsuragi et al., 2015). Plant detection of the many flagellin epitopes appears to rely on different perception systems, including the potential FLS3 receptor (Clarke et al., 2013). At least two of these flagellin perception systems appear to co-exist in rice (Katsuragi et al., 2015), where they would maximize the plant defense strategy and reduce the chance of MAMP evasion. An additional eliciting region has also been identified in the EFa50 domain of the EF-Tu bacterial protein (Furukawa et al., 2014). While the EF-Tu-derived elf18 peptide is only perceived in Brassicaceae species, the recently identified EFa50 domain is fully active in rice and extends the possibility of EF-Tu recognition.

To avoid MTI recognition, bacteria use additional strategies to site mutation of active epitopes. For example, MAMPs can be masked by post-translational modifications, such as flagellin glycosylation (Hirai et al., 2011) (Figure 1C). *Pta* possesses pathovar-specific post-translational modifications to prevent the hypersensitive response (HR)-inducing activity of flagellin in tobacco (Taguchi et al., 2003). Flagellin glycosylation seems ubiquitous for different bacteria (Ichinose et al., 2013) and is required for the virulence of *Pta, P. syringae* pv. *glycinea*, *P. aeruginosa* and *Xcc* on their plant hosts (Taguchi et al., 2003, 2006; Takeuchi et al., 2003; Ichinose et al., 2013) or on a murine model (Arora et al., 2005). To reduce the amount of immunogenic epitopes, bacteria can also regulate their flagellin biosynthesis, express multiple flagellin types, shed or completely lack flagella (Hatterman and Ries, 1989; Ramos et al., 2004) (Figure 1D). The deletion in the flagellar gene cluster is observed in *Xanthomonas fuscans* pv. *fuscans* which is not motile but remains pathogenic on bean (Darrasse et al., 2013). The modulation of flagellum content, depending on the stage of root colonization, was reported in bacteria like *Pseudomonas brassicacearum* (Achouak et al., 2004). Several bacteria even secrete the alkaline proteases AprA, which specifically degrade flagellin monomers, spilled during the flagella construction or damage, into inactive peptides (Bardoel et al., 2011; Pel et al., 2014) (Figure 1E). Such strategy hampers the recognition by both TLR5 and FLS2 (Bardoel et al., 2011). The AprA-mediated MTI evasion seems to be widespread among bacteria species, including the beneficial ones, and leads to enhanced bacterial virulence on both plants (Pel et al., 2014) and animals (Howe and Iglewski, 1984; Liehl et al., 2006). Bacterial strategies to perturb MTI during symbiosis or pathogenesis also rely on secretion of glyco-conjugates, such as cyclic glucan or extracellular polysaccharides (Silipo et al., 2010) (Figure 1F). Finally, the delivery of bacterial toxins (like coronatine or syringolin) or effectors inside the plant host cell is an effective strategy to control MTI (Boller and Felix, 2009). Bacterial effectors have been shown to target *de novo* PRR biogenesis or directly affect the stability and activity of PRRs and their co-receptors (reviewed in Macho and Zipfel, 2014) (Figure 1G).

## Conclusion

Although many studies show that PRRs are key for plant immunity, not all of the PRRs studied so far seem to contribute similarly to plant resistance. Upon plant-pathogen interactions, the importance of a given PRR depends on its level of expression, the abundance of its cognate MAMP, the rapidity and efficiency of the immune activation after ligand binding and last, but not least, on the set of pathogen strategies to bypass that given PRR sensing system.

### Conflict of interest statement

The authors declare that the research was conducted in the absence of any commercial or financial relationships that could be construed as a potential conflict of interest.
