# Supplementary material for: Perception of pathogenic or beneficial bacteria and their evasion of host immunity: pattern recognition receptors in the frontline
Source: Front Plant Sci. 2015 Apr 8;6:219. doi: 10.3389/fpls.2015.00219 (PMC4389352; doi:10.3389/fpls.2015.00219)
Supplement: Supplementary file 1 [file FigureS1.DOCX]

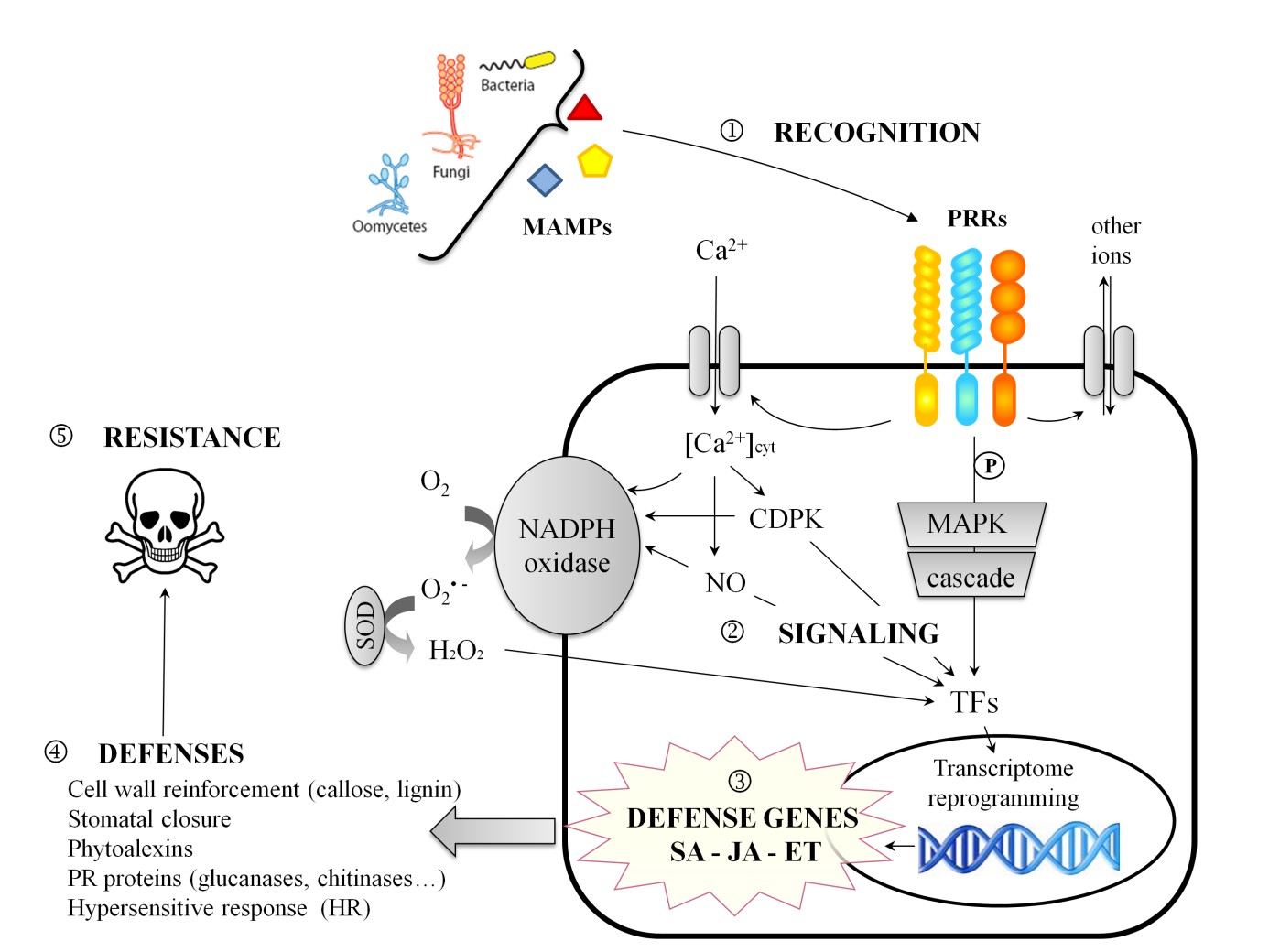


**Figure S1: Schema representing the cascade of events elicited by a MAMP after PRR recognition.** The perception of a MAMP by its cognate PRR (1) elicits a signaling cascade (2) including ion fluxes through the plasma membrane generally leading to an increased cytosolic Ca^2+^ concentration, calcium-dependent protein kinase (CDPK) and mitogen-activated protein kinase (MAPK) activation, H_2_O_2_ and NO production. The integration of these secondary messengers activates transcription factors (TFs) which trigger a transcriptome reprogramming. This leads to the rapid production of phytohormones such as salicylate (SA), jasmonate (JA) or ethylene (ET) and to the expression of defense genes (3) involved in cell wall strengthening, stomatal closure, the production of phytoalexins and hydrolytic pathogenesis-related (PR) proteins and sometimes to a hypersensitive response (HR)-like cell death at the infection site (4). Altogether these defenses contribute to the induced resistance (5).
